# Genetic and Environmental Overlaps Among Sasang Constitution Types: A Multivariate Twin Study

**DOI:** 10.1017/thg.2018.56

**Published:** 2018-10-31

**Authors:** Yoon-Mi Hur, Siwoo Lee, Hee-Jeong Jin

**Affiliations:** 1 Research Institute for the Welfare Society, Mokpo National University, Jeonnam, South Korea; 2 Mibyeong Research Center, Korea Institute of Oriental Medicine, Daejeon, South Korea

**Keywords:** Sasang constitution types, pleiotropy, genes, environment, twin, alternative medicine

## Abstract

According to the Sasang theory, humans can be categorized into one of the four Sasang constitution (SC) types. The four SC types are Tae-Yang (TY), Tae-Eum (TE), So-Yang (SY), and So-Eum (SE), which are determined mainly on the basis of anthropometric characteristics, personality, and the balance of the physiological functions of the major organ systems. There is a growing recognition in the complementary and alternative medicine area that SC types have the potential to be a useful scientific tool for the prevention, diagnosis, and treatment of diseases (Cooper, *Evidence Based Complementary and Alternative Medicine*, Vol. 6 (Suppl. 1), 2009, pp. 1–3). The main purposes of the present study are to estimate genetic and environmental influences on SC types, and to explore genetic and environmental correlations that affect phenotypic associations among the SC types. In total, 1,742 (365 monozygotic male, 173 dizygotic male, 675 monozygotic female, 271 dizygotic female, and 258 opposite-sex dizygotic) twins (mean age = 19.1 ± 3.1 year) completed a Sasang questionnaire. Univariate and multivariate model-fitting analyses were performed. Total (additive and non-additive) genetic influences were 71% for males and 81% for females in TE, 70% for males and 71% for females in SE, and 47% for both sexes in SY. Non-additive genetic effects were substantial, and shared environmental influences were negligible in most SC types. Multivariate model-fitting analysis revealed that non-additive genetic and individual-specific environmental correlations between TE and SE were -0.92 (95% CI [-0.89, -0.93]) and -0.62 (95% CI [-0.57, -0.68]), respectively. The corresponding estimates were -0.55 (95% CI [-0.48, -0.61]) and -0.44 (95% CI [-0.37, -0.51]) between TE and SY and 0.19 (95% CI [0.09, 0.29]) and -0.40 (95% CI [-0.32, -0.47]) between SE and SY. These results suggest that the phenotypic associations among SC types may be mediated by pleiotropic mechanism of genes.

According to the Sasang constitutional (SC) theory originally developed by Lee Je Ma (1837–1900) who specialized in Korean traditional medicine, there are four constitutional types in humans and one of these four types is dominant for a person from early in life (Kim & Pham, 2009; Lee & Choi, 1996). The four SC types are Tae-Yang (TY), Tae-Eum (TE), So-Yang (SY), and So-Eum (SE), which are determined on the basis of body shape (e.g., chest, abdomen, hip, waist, and neck), facial configuration, skin tactility (e.g., elasticity, roughness, and wrinkle density), voice (e.g., pitch, frequency, speed, and resonance), personality characteristics, and the balance of the physiological functions of the major organ systems, especially respiratory (lung), digestive (liver), urinary (kidney), and lymphatic (spleen) systems (Kim & Pham, 2009). Recently, questionnaires and scientifically standardized methods such as computer-based analyses of body shape, 3D facial pictures and voice records have been developed to objectively measure SC types as well as to validate the Sasang theory (Cooper, 2009; Lee et al., 2009a).

Table 1 shows major characteristics of the four SC types. For the TY type, the respiratory system tends to function prominently. The TY type tends to have slender waist and be creative, positive, and heroic. However, the TY type is extremely uncommon (0.1%) in many populations (Chae et al., 2003). For this reason, TY has often been excluded from empirical research. The TE type tends to have a large waist circumference, perspire freely, and be cautious, gentle, and patient. For the TE type, the function of the digestive system tends to be prominent. The SY type is characterized by large chest, small hip, and good bowel movement. The function of the lymphatic system is prominent for the SY type. In terms of personality, the SY type tends to be hot-tempered, and easily bored (high novelty seeking). The urinary system functions prominently in SE. The SE type tends to have large hips and a small chest, and is characterized by increased harm avoidance, nervousness, and conscientiousness (Chae et al., 2003).

**TABLE 1:** Major Characteristics, and an Informal Review of Studies of Prevalence of Diseases and GWAS of TY, TE, SY, and SE Published in English in Peer-Reviewed Journals

Characteristics	TY	TE	SY	SE
Physique	Slender waist	Obese, large waist circumference	Small hip, large chest	Large hip, small chest
The prominently functioning organ system	Respiratory	Digestive	Lymphatic	Urinary
Personality	Creative, positive, heroic	Cautious, gentle, patient	High novelty seeking, hot-tempered	High harm-avoidance, nervous, conscientious
Prevalence of diseases		Obesity (Baek et al., 2014); Insulin resistance (Choi et al., 2011); Diabetes mellitus (Lee et al., 2009a); Hypertension (Lee et al., 2011); Lower prevalence of cancers (Lee et al., 2013); Functional dyspepsia in females (Kim et al., 2015)		Functional dyspepsia in males (Kim et al., 2015)
GWAS		3q27.3 (rs10937331), *GPM6A, SYT4, GRIK1, LPP9*, and *CACNA1A* genes (Kim et al., 2012); APOC1, C2orf47 (Kim et al., 2017)	14q22.3 (rs12431592), *ZFP42, CDH22, ALDH1A2, OTX2, EN2* genes (Kim et al., 2012)	15q22.2 (rs7180547), *AKAP11, PTPN2, NRP2* genes (Kim et al., 2012); FTO (rs7193144) (Cha et al., 2015);

Note: TY = Tae-Yang; TE = Tae-Eum; SY = So-Yang; SE = So-Eum; GWAS = genome-wide association studies.

Several large-scale studies have demonstrated that prevalence rates and risks of diseases vary across different SC types, suggesting that susceptibilities to diseases may differ across constitution types (Table 1). For example, as compared to SE or SY, TE has been shown to have a significantly higher prevalence in metabolic disorders and cardiovascular diseases, including obesity (Baek et al., 2014), abdominal obesity (Baek et al., 2014; Jang et al., 2013), insulin resistance (Choi et al., 2011), diabetes mellitus (Lee et al., 2009b) and hypertension (Lee et al., 2011). Song et al. (2012) also found that serum high-density lipoprotein (HDL) cholesterol and higher triglyceride levels were higher in TE than in other constitution types, suggesting that higher prevalence in metabolic and cardiovascular diseases in TE may be due in part to elevated lipid traits for the TE type. On the other hand, Lee et al. (2013) reported that TE had lower rates of various types of cancers as compared to other SC types. Kim et al. (2015) showed sex differences in the prevalence of functional dyspepsia: While the prevalence was higher in SE than in non-SE in males, it was higher in TE than in non-TE in females.

Although twin studies on SC types have been rarely conducted to date, SC researchers have long speculated that SCs are inherited characteristics (Shim et al., 2008). Recently, genome-wide association studies (GWAS) have been performed to identify genes for each SC type in the South Korean population (Table 1). For example, after genotyping over 1,000 subjects classified by the SC type, Kim et al. (2012) found that chromosomes 3q27.3 (rs10937331), 15q22.2 (rs7180547), and 14q22.3 (rs12431592) were most significantly associated with TE, SE, and SY respectively. They further noted that neuron-related genes such as *GPM6A*, *SYT4*, *GRIK1*, *LPP9*, and *CACNA1A* were significantly associated with TE; cell-signaling genes, *AKAP11, PTPN2*, and *NRP2* were associated with SE; and that 17 other genes, including *ZFP42*, *CDH22*, *ALDH1A2*, *OTX2*, and *EN2* were associated with SY. Subsequently, Cha et al. (2015) found in a GWAS (*N* = 3,810) that the fat mass and obesity-associated (FTO) variant (rs7193144) was significantly inversely related to the SE type after adjusting for body mass index (BMI). This finding was replicated in an independent sample (*N* = 1,680; Cha et al., 2015). Using a sample of over 5,000 South Koreans, Kim et al. (2017) performed a GWAS and reported that two gene loci, APOC1 on chromosome 19 and C2orf47 were significantly associated with the ratio of LDL and HDL cholesterol only in the TE but not in the non-TE types. As the lipid cholesterol ratio is known to be a risk factor for cardiovascular diseases and metabolic syndrome (Ingelsson et al., 2007), these findings were in line with prior phenotypic studies that reported higher prevalence of metabolic and cardiovascular disorders in TE than in non-TE (Song et al., 2012).

Assuming that SC types are independent categories (Kim & Pham, 2009), many SC researchers to date have sought to identify vulnerabilities to diseases and genes unique to each SC type. However, as the four SC types reflect complex human physical, physiological, and psychological aspects that are known to be influenced largely by multiple genes with small effects and pleotropic mechanisms of the genes (Solovieff et al., 2013), it is likely that there are genetic commonalities as well as uniqueness among the four SC types. The aim of the present study was therefore to explore the phenotypic relationships of the SC types and genetic and environmental overlaps that may give rise to these phenotypic associations. To this end, we used a SC questionnaire recently developed under the assumption that underlying liability of each SC type is continuous (Baek et al., 2015).

## Methods

### Sample

The sample consisted of 1,742 (365 monozygotic [MZ] male, 173 dizygotic [DZ] male, 675 MZ female, 271 DZ female, and 258 opposite-sex DZ) twins who participated in the 2017 survey conducted at the South Korean Twin Registry (Hur et al., 2013). The age of the sample at the time of the assessment ranged from 11 years to 29 years with a mean of 19.1 years (*SD* = 3.1 years). Twins under 20 years of age were recruited mostly from schools throughout South Korea, while those 20 years of age or older were from Facebook, twin clubs on the internet, and colleges throughout South Korea. Further details of the sample collection are described in Hur et al. (2018).

Zygosity of the twins was assessed using a three-item zygosity questionnaire. When compared to DNA analysis, this approach has been shown to achieve over 90% accuracy (Ooki et al., 1990). The number of MZ twins was much greater than that of DZ twins in the present sample, which likely reflected the low DZ twin birth rates in the South Korean population for the birth cohorts in the present study (Hur & Kwon, 2005; Hur & Song, 2009).

### Measure

Trained interviewers gave twins the Korean SC Diagnostic Questionnaire-15 (KS-15; Table 2) via a telephone interview. The KS-15 was developed and validated using health information about subjects (*N* = 1,867) registered with the Korea Constitutional Multicenter Bank (Baek et al., 2015). The KS-15 consists of items on current height and weight, six personality questions, and eight symptom questions regarding physiological functions of internal organs following the SC theory. Using height and weight, BMI was calculated to determine the level of obesity of the respondent after adjustment for sex and age. Each of the six personality and eight symptom questions includes three options, and respondents are instructed to select one of the three options that best describes them. During the development of the KS15, clinicians specialized in SC assigned a weight to each of the three options through consensus so that the scores of TE, SE, and SY can be computed by summing the weighted responses for each type. The resulting scores of TE, SE, and SY ranged from 0 to 1.0. We did not score TY because the scoring scheme was not developed for the TY type in the KS-15. The items of the KS15 have been demonstrated to have adequate psychometric properties (Kim & Jang, 2016).

**TABLE 2:** Items of the Korean Sasang Constitution Diagnostic Questionnaire-15

	Item content
1	Current height (cm); current weight (Kg)
2	Broad-minded?
3	Act quickly?
4	Active, enthusiastic?
5	Extraverted?
6	Masculine or feminine?
7	Irrational, easily upset?
8	Digest well?
9	Good appetite?
10	Sweat a lot?
11	Feeling after sweat
12	Rectal urgency (unable to hold stool during bowel urgency)
13	Wake up at night for urination? If so, how often?
14	Aversion to heat or cold?
15	Preference for warm or cold water?

### Statistical Analyses

Twin studies make use of the difference in the proportion of genes shared between MZ and DZ twins. Utilizing this difference, the total variance of each SC type is decomposed into following components: additive genetic (A: the effects of individual genes summed over loci, *r*_MZ_ = 1.0; *r*_DZ_ = 0.5), non-additive genetic (D: the interactive effects of alleles across loci or within a locus, *r*_MZ_ = 1.0; *r*_DZ_ = 0.25), shared environment (C: the effects of environment that make twins similar, *r*_MZ_ = 1.0; *r*_DZ_ = 1.0), and individual-specific environment plus measurement error (E: the effects of environment unique to each member of a twin pair that decrease twin similarity, *r*_MZ_ = 0; *r*_DZ_ = 0).

Data analyses consisted of univariate and multivariate components. In the univariate analysis, we computed maximum likelihood correlations for five twin groups (MZ males, DZ males, MZ females, DZ females, and opposite-sex DZ) and carried out general-sex limitation model-fitting analyses for each SC type to obtain an initial indication on genetic and environmental estimates for each SC type. In the univariate model-fitting analysis we compared the ACE and ADE model for each SC type by using Akaike's information criterion (AIC; Akaike, 1987). The lower the value of AIC, the better the balance between explanatory power and parsimony (Akaike, 1987). We ran the ACE and the ADE models separately because non-additive genetic effects are difficult to distinguish from shared environmental effects in the twin design (Neale & Cardon, 1992).

In the multivariate analysis, we computed phenotypic correlations among three SC types in the total sample, MZ and DZ cross-twin, cross-trait correlations, and conducted a Cholesky decomposition model-fitting analysis. As multivariate analysis requires a large sample especially in the presence of non-additive genetic effects, prior to computing cross-twin, cross-trait correlations and completing Cholesky decomposition model-fitting analyses, we combined males and females and adjusted for sex, age, age × sex, and age^2^ by using a regression procedure (McGue & Bouchard, 1984).

Cholesky decomposition model divided the phenotypic associations among the three SC types into the proportion of variance associated with additive genetic, non-additive genetic, shared environment, and individual-specific environment factors. The additive genetic, non-additive genetic, shared environment, and individual-specific environment covariance matrices were computed by the product of their respective Cholesky factor loading matrix and its transpose. The additive genetic (*r*_g_), non-additive genetic (*r*_d_), shared environmental (*r*_c_), and individual-specific environmental correlations (*r*_e_) among the three scales were also derived from these variances and covariances. The additive genetic correlation provides a measure of the extent to which two SC types are influenced by the same genes that operate additively. For example, if the additive genetic correlation is estimated at 1.0 between TE and SE, this indicates that TE and SE share all of their additive genetic factors; if this correlation is estimated at zero, then there are no shared additive genetic factors between TE and SE. The same logic applies to non-additive genetic, shared environment, and individual-specific environmental correlations.

We used a raw data option in Mx (Neale et al., 2003) to carry out model-fitting analysis. Mx yields twice the negative likelihood (-2LL) of the models, which is distributed as a chi-square. The fit of the full Cholesky model was compared to that of nested submodels by using the difference in twice the negative log-likelihood (-2LL) of the models, which is distributed as a chi-square. A significant (*p* < .05) change in chi-square in the nested submodel indicates that removing the parameters from the full model significantly worsens the model-fit, and therefore the submodel is rejected.

## Results

### Descriptive Statistics

The distribution of SY was fairly normal with a skewness of -0.11. TE and SE were moderately positively skewed with skewness indices of 0.62 and 0.82, respectively. We thus performed square root transformation of the data for TE and SE, which resulted in skewness indices of 0.05 and 0.02, respectively. Table 3 presents mean (*SD*) and correlations with age for TE, SE, and SY. Age was very modestly correlated with all three types (-0.09 < *r* < 0.05) in our sample, suggesting that the main effect of age in SC types may be negligible in adolescents and young adults. Males were significantly higher than females in TE (*t* = 7.42, *p* < 0.001). In SE and SY, however, females were significantly higher than males (*t* = -4.47, *p* < .001 for SE; *t* = -6.32, *p* < .001 for SY).

**TABLE 3:** Mean and Correlation With Age (*r*) for TE, SE, and SY

	TE		SE		SY	
	Mean (*SD*)	*r*	Mean (*SD*)	*r*	Mean (*SD*)	*r*
Male	0.44 (0.26)	-0.09*	0.23 (0.20)	0.05	0.33 (0.17)	0.08*
Female	0.35 (0.24)	-0.05	0.27 (0.20)	0.03	0.38 (0.14)	0.05

Note: *p < .05. TE = Tae-Eum; SY = So-Yang; SE = So-Eum.

### Twin Correlations and Univariate Model Fitting

**FIGURE 1.**
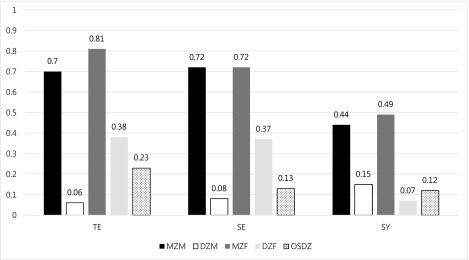
Maximum likelihood twin correlations for TE (Tae-Eum), SE (So-Eum), and SY (So-Yang) by sex and zygosity.

Figure 1 presents twin correlations for three SC types by zygosity and sex. For all three types, MZ twin correlations were significantly greater than DZ twin correlations in both sexes, suggesting the importance of genetic influences on SC. Furthermore, DZ twin correlations were less than half the MZ twin correlations for all three types, indicating that shared environmental influences are negligible, whereas genetic non-additivity is important. DZ twin correlations were lower in males than in females for TE and SE, indicating that non-additive genetic effects may be greater in males than in females for these two types. MZ twin correlations were greater in SE and TE than in SY in both sexes, which indicated that genetic influences are greater in SE and TE than in SY.

Table 4 presents the results of univariate model-fitting analyses, including parameter estimates from the full ACE and the ADE models for TE, SE, and SY. For all three SC types, the ADE model yielded a lower AIC value than did the ACE model, suggesting that ADE explained the data better than did ACE, consistent with observations made from twin correlations. Sex-specific genetic effects were not significant in any of the SC types. However, non-additive genetic effects were predominant in males, whereas additive genetic effects were substantial in females in TE and SE. The total genetic influences (additive and non-additive genetic effects) in the full ADE models were 71% for males and 81% for females in TE, 70% for males and 71% for females in SE, and 47% for both sexes in SY. Non-genetic variances were due to individual-specific environmental influences plus measurement error.

**TABLE 4:** Results of Univariate Model-Fitting Analysis for TE, SE, and SY

					Male				Female			
Variable	Model	-2LL	AIC	*df*	A	C	D	E	A	C	D	E
TE	ACE	-833.3	-3867.4	1517	0.66 [0.48, 0.76]	0.03 [0, 0.20]	–	0.31 [0.23, 0.40]	0.72 [0.51, 0.83]	0.09 [0.0, 0.29]	–	0.19 [0.16, 0.23]
	**ADE**	**-839.4**	**-3873.4**	**1517**	**0.10 [0.0, 0.48]**	**–**	**0.61 [0.21, 0.76]**	**0.29 [0.23, 0.38]**	**0.71 [0.07, 0.84]**	**–**	**0.10 [0.0, 0.74]**	**0.19 [0.16, 0.23]**
SE	ACE	-559.0	-3593.0	1517	0.66 [0.48, 0.76]	0.03 [0.0, 0.20]	–	0.30 [0.24, 0.39]	0.55 [0.32, 0.73]	0.16 [0.0, 0.38]	–	0.29 [0.25, 0.35]
	**ADE**	**-564.3**	**-3598.3**	**1517**	**0.07 [0.0, 0.13]**	**–**	**0.63 [0.57, 0.76]**	**0.30 [0.23, 0.38]**	**0.69 [0.09, 0.76]**	**–**	**0.02 [0.0, 0.62]**	**0.29 [0.24, 0.34]**
SY	ACE	-1576.0	-4610.0	1517	0.22 [0, 0.47]	0.21 [0.01, 0.46]	–	0.58 [0.46, 0.74]	0.37 [0.14, 0.56]	0.08 [0.0, 0.29]	–	0.55 [0.46, 0.64]
	**ADE**	**-1583.5**	**-4617.5**	**1517**	**0.20 [0, 0.56]**	**–**	**0.27 [0.0, 0.57]**	**0.53 [0.44, 0.67]**	**0.00 [0.0, 0.51]**	**–**	**0.47 [0.0, 0.55]**	**0.53 [0.45, 0.61]**

Note: TE = Tae-Eum; SY = So-Yang; SE = So-Eum. -2LL = -2 log likelihood; A = additive genetic effects, C = shared environmental effects, D = non-additive genetic effects, E = individual-specific environmental effects plus measurement error. 95% CIs are shown in square brackets. Best-fitting models are indicated in bold.

### Phenotypic and Cross-Twin, Cross-Trait Twin Correlations, and Multivariate Model Fitting

Table 5 presents phenotypic, cross-twin cross-trait correlations for MZ and DZ twins for TE, SE, and SY. Phenotypic correlations between TE and SE and between TE and SY were negative and high (*r* = -0.89 and *r* = -0.48), indicating the phenotypic overlap. Furthermore, the directions of the phenotypic correlations were consistent with those of the MZ and DZ cross-twin, cross-trait correlations, suggesting that the direction of effect may be the same. Because common shared environmental influences were negligible, the MZ cross-twin, cross-trait correlation represented genetic sources of covariance. Thus, greater MZ than DZ cross-twin, cross-trait correlations in Table 5 indicated the presence of substantial genetic overlaps between TE and SE and between TE and SY.

**TABLE 5:** Phenotypic and Cross-Twin Cross-Trait Correlations for MZ and DZ Twins for TE, SE, and SY

		Cross-twin, cross-trait	
		correlation	
Variable	Phenotypic r	MZ	DZ
TE-SE	−0.89**	−0.69**	−0.22**
TE-SY	−0.48**	−0.30**	−0.12*
SE-SY	0.15**	−0.10	−0.08

Note: MZ = monozygotic twin, DZ = dizygotic twins.*p < .05, **p < .01. TE = Tae-Eum; SY = So-Yang; SE = So-Eum.

A small but significant phenotypic correlation was found between SE and SY (*r* = 0.15, *p* < .01). Although the MZ cross-twin, cross-trait correlation for the relationship between SE and SY was slightly greater than the corresponding DZ correlation, both correlations were low, indicating that individual-specific environment may play a role in the relationship between SE and SY.

**FIGURE 2.**
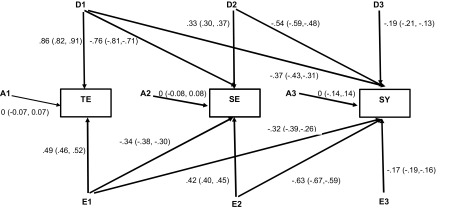
Parameter estimates (95%CI) for TE (Tae-Eum), SE (So-Eum), and SY (So-Yang) in the best-fitting Cholesky model. Note: Unstandardized additive genetic (A1, A2, and A3), non-additive genetic (D1, D2, and D3), and individual-specific environmental (E1, E2, and E3) path coefficients in the best-fitting Cholesky model for TE, SE, and SY. Path coefficients can be squared to obtain variance associated with each factor.

Table 6 shows the results of Cholesky decomposition model-fitting analysis and Figure 2 presents parameter estimates in the best-fitting Cholesky model. Consistent with the results of univariate model-fitting analysis, the ADE model yielded lower AIC than did the ACE model, indicating that the ADE model fit the data better than the ACE model (Model 1 vs. Model 2). Thus, the fits of the submodels were compared to those of the ADE rather than the ACE model. Although additive genetic covariances could be removed from the ADE model without significantly worsening the model-fit (Model 4), non-additive genetic or environmental covariances could not (Models 5 and 6). From these comparisons, Model 4 was chosen as the best fitting one.

**TABLE 6:** The Results of Cholesky Decomposition Model-Fitting Analysis

Model	Model description	-2LL	AIC	*df*	Δ-2ll	Δ*df*	*p*
1	ACE	7,366.5	-3037.5	5,202			
2	ADE	7,345.9	-3058.1	5,202			
3	AE	7,366.5	-3049.5	5,208	20.6	6	.00
**4**	**ADE without additive genetic covariances**	**7,347.1**	**-3062.9**	**5,205**	**1.3**	**3**	**.74**
5	ADE without non-additive genetic covariances	7,356.0	-3054.0	5,205	10.1	3	.02
6	ADE without environmental covariances	11,406.9	996.9	5,205	4,061.0	3	.00

Note: -2LL = -2 log likelihood; A = additive genetic effects, C = shared environmental effects, D = non-additive genetic effects, E = individual-specific environmental effects plus measurement error. Best-fitting model is indicated in bold.

As can be seen in Figure 2, non-additive genetic and individual-specific environmental variances and covariances among three SC types were all significant, while all of the additive genetic variances were estimated as zero. These results indicated the presence of significant non-additive genetic and individual-specific environmental overlaps that contributed to phenotypic associations among three SC types. Table 7 presents total genetic and individual-specific environmental variance estimates for TE, SE, and SY, and non-additive genetic and individual-specific environmental correlations derived from the parameter estimates in Figure 2. Total genetic variance estimates were 76%, 70%, and 47% for TE, SE, and SY respectively, which were close to the averages of the total genetic effects in males and females in the ADE univariate models in Table 3. The non-additive genetic correlation between TE and SE was very high (*r* = -0.92) and greater than individual-specific environmental correlation (*r* = -0.62). The non-additive genetic correlation between TE and SY was moderate but still greater than the corresponding individual-specific environmental correlation (*r* = -0.55 vs. *r* = -0.44). Taken together, these results provide evidence for significant genetic contributions to the phenotypic associations between TE and SE and between TE and SY.

**TABLE 7:** Total Genetic and Individual-Specific Environmental Variance Estimates and Correlations and Their 95% Confidence Intervals Derived From the Best-Fitting Cholesky Model

	Variance estimates				
Variable	Total genetic	Environmental	Relationship	*r*_d_	*r*_e_
TE	76% [72%, 79%]	24% [21%, 28%]	TE-SE	-0.92 [-0.89, -0.93]	-0.62 [-0.57, -.68]
SE	70% [66%, 74%]	30% [26%, 34%]	TE-SY	-0.55 [-0.48, -0.61]	-0.44 [-0.37, -0.51]
SY	47% [40%, 53%]	53% [47%, 60%]	SE-SY	0.19 [0.09, 0.29]	-0.40 [-0.32, -0.47]

Note: *r*_d_ = non-additive genetic correlation, *r*_e_ = individual-specific environmental correlation. 95% CIs are shown in square brackets.

While the non-additive genetic correlation between SE and SY was positive, the individual-specific environmental correlation was negative and larger than the non-additive genetic correlation. As the phenotypic correlation between SE and SY was positive, this result suggested that the effects of common genes may be strong enough to offset common environmental effects on the relationship between SE and SY. One should note that genetic correlations reflect the amount of the genetic influences on two traits that is common to both. This is independent of the heritability of either trait. Therefore, it is possible for a trait to have a small genetic correlation with another trait but to have a high heritability and make a high contribution to the phenotypic association (Plomin et al., 2012, p. 397).

## Discussion

To date, little research attention has been given to pleiotropic associations among the SC types. Pleiotropy is the overlap between the genetic architecture of two or more traits due to a variety of shared causal pathways (Solovieff et al., 2013). In the present study we conducted multivariate genetic analyses to determine genetic and environmental overlaps among three SC types: TE, SE, and SY. We first found that genetic non-additivity significantly contributed to individual difference in each SC type. Second, all phenotypic associations among the SC types were significant, suggesting that SC types are not independent. Finally, Cholesky model-fitting analysis revealed that these phenotypic associations were largely due to non-additive genetic correlations among the SC types, although individual-specific environmental correlations also contributed to the associations.

High heritability estimates for the SC types, especially for TE and SE found in this study, pointed to the importance of further identification of novel genetic loci and biological pathways contributing to the heritability. Extreme skewness of the raw distribution can sometimes generate scale-dependent evidence for genetic non-additivity (Jinks & Fulker, 1970). However, prior to twin analysis, we removed skewness by transforming the data. Thus, significant non-additive genetic variances found in the present study suggested that gene × gene interactions may be an important source of individual difference in SC types, that SC types may have complex polygenic nature, and that large sample sizes are likely to be required to identify specific genetic variants and their interactions. Although the magnitudes of total genetic influences on TE and SE were similar in males and females, the nature of genetic effects (additive vs. non-additive) were different between two sexes in TE and SE. Replications of our findings with a larger sample may be needed to draw a firm conclusion on sex-difference in additive versus non-additive genetic effects in TE and SE. Non-additive genetic variance in SC types may in part reflect genetic effects in personality traits because the KS15 includes six personality items (Table 1). Several twin studies have demonstrated that non-additive genetic effects were significant in the variations of personality (Hur, 2007; Keller et al., 2005). Multivariate genetic analyses on personality and SC types in the future may help to elucidate genetic and environmental links between SC types and personality dimensions.

Non-additive genetic correlations were significant and large between TE and SE and between TE and SY, indicating that genes that influence TE may affect SE and SY as well. Especially, non-additive genetic correlation of -0.92 between TE and SE suggested that nearly identical genetic factors may influence TE and SE. However, negative signs of the genetic correlations suggest that genes that increase TE may suppress the effects of SE and SY. For better understanding of underlying mechanisms for the phenotypic associations, future molecular genetic studies should test for pleiotropy using SNP-based genetic data.

Although individual-specific environmental correlations between TE and SE and between TE and SY were not as large as corresponding genetic correlations, they were significant, suggesting that common sets of environment may also mediate the relationships of these phenotypes. Similar to genetic correlations, the negative signs of individual-specific environmental correlations indicated that environmental sources that enhance TE may reduce SE and SY and vice versa. For example, certain nutrition may help to develop the TE type but suppress the development of the SE type. One should note that correlated measurement errors are confounded with individual-specific environmental correlations. Thus, actual individual-specific environmental correlations may be lower than those estimated in our study.

Our study has some limitations. First, non-additive genetic correlations in our study should be interpreted as a sum of additive and non-additive genetic correlations as it is likely that our sample size was not large enough to separate between the two types of correlations. Second, we only used the questionnaire method to measure SC types. Future studies should employ multiple measures and conduct multivariate genetic analyses to determine genetic sources common across measures and those unique to each measure. Third, as the order of the variables in the Cholesky decomposition model is arbitrary, we changed the order of the variables in various ways prior to Cholesky model fitting. However, the results of analyses were the same. That is, Cholesky models with different orders of the variables yielded the same values of the goodness-of-fit statistics, heritability estimates, and genetic and environmental correlations. Finally, our sample was based on South Korean adolescents and young adults. Our results therefore need to be replicated in larger samples with different ethnic and genetic backgrounds.
